# A strategy of designing high-entropy alloys with high-temperature shape memory effect

**DOI:** 10.1038/s41598-019-49529-8

**Published:** 2019-09-11

**Authors:** Je In Lee, Koichi Tsuchiya, Wataru Tasaki, Hyun Seok Oh, Takahiro Sawaguchi, Hideyuki Murakami, Takanobu Hiroto, Yoshitaka Matsushita, Eun Soo Park

**Affiliations:** 1 International Center for Young Scientists, National Institute for Materials Science, 1-2-1 Sengen, Tsukuba, Ibaraki 305-0047 Japan; 2 Research Center for Structural Materials, National Institute for Materials Science, 1-2-1 Sengen, Tsukuba, Ibaraki 305-0047 Japan; 3 Graduate School of Pure and Applied Sciences, University of Tsukuba, 1-1-1 Tenodai, Tsukuba, Ibaraki 305–8577 Japan; 4 Research Institute of Advanced Materials, Department of Materials Science and Engineering, Seoul National University, Seoul, 08826 Republic of Korea; 5 Department of Nanoscience and Nanoengineering, Graduate School of Advanced Science and Engineering, Waseda University, 3-4-1, Okubo, Shinjuku-ku 169-8555 Japan; 6 Materials Analysis Station, National Institute for Materials Science, 1-2-1 Sengen, Tsukuba, Ibaraki 305-0047 Japan; 7 Present Address: School of Materials Science and Engineering, Pusan National University, Busan, 46241 Republic of Korea

**Keywords:** Mechanical properties, Metals and alloys

## Abstract

Shape memory effect, the ability to recover a pre-deformed shape on heating, results from a reversible martensitic transformation between austenite and martensite phases. Here, we demonstrate a strategy of designing high-entropy alloys (HEAs) with high-temperature shape memory effect in the CrMnFeCoNi alloy system. First, we calculate the difference in Gibbs free energy between face-centered-cubic (FCC) and hexagonal-close-packed (HCP) phases, and find a substantial increase in thermodynamic equilibrium temperature between the FCC and HCP phases through composition tuning, leading to thermally- and stress-induced martensitic transformations. As a consequence, the shape recovery temperature in non-equiatomic CrMnFeCoNi alloys can be increased to 698 K, which is much higher than that of conventional shape memory alloys (SMAs) and comparable to that of B2-based multi-component SMAs containing noble metals (Pd, Pt, *etc*.) or refractory metals (Zr, Hf, *etc*.). This result opens a vast field of applications of HEAs as a novel class of cost-effective high-temperature SMAs.

## Introduction

The concept of high-entropy alloys (HEAs) and complex, concentrated alloys (CCAs), which consist of multi-principal elements with near equiatomic composition, have generated substantial interest for the exploration of immense composition space offered by multiple principal elements and the development of new materials with exceptional properties^1–5^. Cr_20_Mn_20_Fe_20_Co_20_Ni_20_ alloy (in at.%), with a simple lattice of FCC crystal structure, is one of the most extensively studied single-phase HEAs^6–13^. At low temperatures, this alloy exhibits excellent fracture toughness and tensile properties due to deformation-induced nano-twinning, which is favored in low stacking fault energy materials^11–15^. Theoretical approaches predict that Gibbs free energy between the FCC and HCP phases (Δ*G*^HCP-FCC^) in the CrMnFeCoNi alloy system can be widely changed through tuning of the alloy composition^15–17^. For example, the Δ*G*^HCP-FCC^ calculation has been used to design metastable dual-phase HEAs with an excellent combination of strength and ductility, which benefit from the massive solid solution strengthening of a HEA and transformation-induced plasticity effect of a metastable FCC phase^16–19^. Other metastable alloys like FeMn-based alloys^20,21^, β-Ti alloys^22^, or β-Mg alloys^23^, have shown their potential for the shape memory effect and superelasticity, which are attributed to reversible martensitic transformations. Although these types of functional applications were recently investigated in B2-based multi-component alloys with increased configurational entropy^24–27^, very little attention has been paid to the shape memory effect in 3*d* transition metal-based HEAs (3*d* HEAs), which generally exhibit better deformability and lower processing cost compared to previously developed shape memory alloys (SMAs).

Here, we develop 3*d* HEAs exhibiting an appreciable shape memory effect in a wide composition range and characterize their shape memory properties. First, we calculate Δ*G*^HCP-FCC^ and thermodynamic equilibrium temperature, *T*_0_, in the various non-equiatomic CrMnFeCoNi HEAs and evaluate the composition dependence of Δ*G*^HCP-FCC^ using CALPHAD methodology with the TCHEA3 database. Second, we carefully reveal the reversible martensitic transformation between the FCC and HCP phases in the compositionally tuned 3*d* HEAs using *in situ* X-ray diffraction (XRD) and thermal analyses. Third, we confirm the shape recovery on heating after pre-deformation in the developed non-equiatomic CrMnFeCoNi HEAs. Indeed, we can manipulate the shape memory effect of the HEAs to have exceptionally wide range of transformation temperatures such as reverse transformation finish temperature from 435 to 698 K. These findings suggest that non-equiatomic CrMnFeCoNi HEAs are promising candidates for cost-effective shape memory actuators even at elevated temperatures.

## Results

### Design of high-entropy alloys with shape memory effect

**Figure 1:**
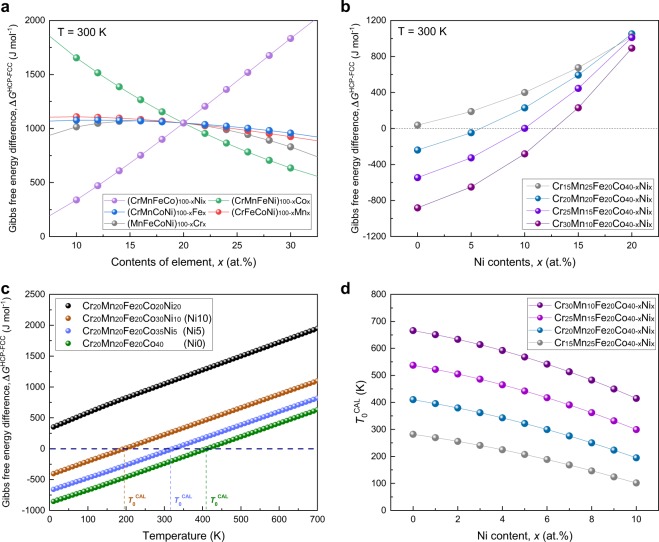
Alloy design for HEAs with shape memory effect. (**a**) CALPHAD predictions of the relative effect of each principal element on the difference in Gibbs free energy between the FCC and HCP phases (Δ*G*^HCP-FCC^) at 300 K in the CrMnFeCoNi alloy system, (**b**) Δ*G*^HCP-FCC^ at 300 K for a series of non-equiatomic CrMnFeCoNi alloys with different Cr/Mn ratios as a function of Ni content, (**c**) temperature dependence of Δ*G*^HCP-FCC^ in a Cr_20_Mn_20_Fe_20_Co_40-x_Ni_x_ system (x = 0, 5, 10, and 20 at.%), and (**d**) *T*_0_^CAL^, the temperature where Δ*G*^HCP-FCC^ is zero in the CALPHAD prediction, for the series of non-equiatomic CrMnFeCoNi alloys with different Cr/Mn ratios.

Figure 1a shows the predicted Δ*G*^HCP-FCC^ at 300 K using CALPHAD methodology with the TCHEA3 database in five hypothetical alloy systems of 3*d* HEAs: (MnFeCoNi)_100-x_Cr_x_, (CrFeCoNi)_100-x_Mn_x_, (CrMnCoNi)_100-x_Fe_x_, (CrMnFeNi)_100-x_Co_x_, and (CrMnFeCo)_100-x_Ni_x_. To evaluate the relative influence of each principal element on Δ*G*^HCP-FCC^, the atomic fraction of the fifth element was modified between 10 and 30 at.% while that of other four elements was kept in equimolar ratio. The CALPHAD prediction showed that Δ*G*^HCP-FCC^ can be significantly reduced when the Ni content decreases or the Co content increases from the equiatomic composition, suggesting that Δ*G*^HCP-FCC^ is significantly reduced by the replacement of Ni with Co. Figure 1b shows Δ*G*^HCP-FCC^ for a series of non-equiatomic CrMnFeCoNi alloys with different Cr/Mn ratios from 0.6 to 3, which were predicted to form a single-phase FCC solid solution^28^. All the alloy series exhibited a pronounced decrease in Δ*G*^HCP-FCC^ as the Ni content was replaced with Co. With an increase in the Cr/Mn ratio up to 3, a more drastic decrease in Δ*G*^HCP-FCC^ was predicted in the alloys with a lower Ni/Co ratio. Compared with Cr_20_Mn_20_Fe_20_Co_20_Ni_20_ alloy, the value of Δ*G*^HCP-FCC^ in several compositions was calculated to be negative. These results imply that the HCP phase can be favored over the FCC phase at 300 K in a wide composition range of non-equiatomic CrMnFeCoNi HEAs.

Figure 1c shows the temperature dependence of Δ*G*^HCP-FCC^ in the Cr_20_Mn_20_Fe_20_Co_40-x_Ni_x_ system (x = 0, 5, 10, and 20 at.%). The Cr_20_Mn_20_Fe_20_Co_20_Ni_20_ alloy showed a positive value of Δ*G*^HCP-FCC^ in the considered temperature range, while the other alloys exhibited a negative value of Δ*G*^HCP-FCC^ at lower temperatures. By replacing Ni with Co in the Cr_20_Mn_20_Fe_20_Co_40-x_Ni_x_ system, *T*_0_^CAL^, the temperature where Δ*G*^HCP-FCC^ is zero in the CALPHAD prediction, increased from 195 to 410 K for Cr_20_Mn_20_Fe_20_Co_30_Ni_10_ and Cr_20_Mn_20_Fe_20_Co_40_ alloys, respectively. Figure 1d shows the predicted *T*_0_^CAL^ for the series of the CrMnFeCoNi alloy system shown in Fig. 1b. As the Ni/Co ratio was reduced, *T*_0_^CAL^ increased from 102 K for the Cr_15_Mn_25_Fe_20_Co_30_Ni_10_ alloy and to 666 K for the Cr_30_Mn_10_Fe_20_Co_40_ alloy. The range of *T*_0_^CAL^ was wider than the range of the experimentally measured *T*_0_ in conventional SMAs such as TiNi (227–359 K)^29^, CuAlNi (241–407 K)^30^, and FeMnSi (363–493 K)^31,32^. These predictions suggest the possibility of developing non-equiatomic CrMnFeCoNi HEAs for functional applications employing the shape memory effect (or pseudoelasticity) since the martensitic transformation from the FCC to HCP phase could occur by undercooling below *T*_0_^CAL^.

**Figure 2:**
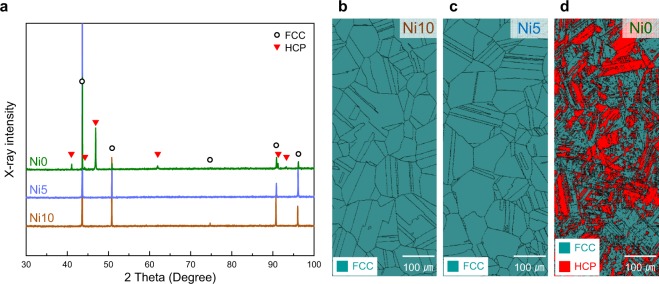
Microstructures of as-annealed 3*d* HEAs. (**a**) XRD patterns of as-annealed alloys produced by induction melting, multi-pass caliber rolling, and annealing at 1373 K for 1 h, exhibiting a single-phase FCC structure in the Ni10 and Ni5 alloys, and a dual-phase FCC-HCP structure in the Ni0 alloy. (**b**,**c**) EBSD phase map of (**b**) Ni10 and (**c**) Ni5 alloys, showing an equiaxed grain structure with an average grain size of about 100 μm. (**d**) EBSD phase map of Ni0 alloy, showing the dual-phase microstructure with HCP fraction of about 40 vol. %.

To understand the relationship among the increase in *T*_0_^CAL^, microstructure, and martensitic transformation behavior in the non-equiatomic CrMnFeCoNi HEAs, we prepared Cr_20_Mn_20_Fe_20_Co_30_Ni_10_ (Ni10), Cr_20_Mn_20_Fe_20_Co_35_Ni_5_ (Ni5), and Cr_20_Mn_20_Fe_20_Co_40_ (Ni0) alloys, shown in Fig. 1c. These HEAs were produced by high-frequency vacuum induction melting and subjected to multi-pass caliber rolling into bars of roughly 14 mm width and annealed at 1373 K for 1 h. XRD (Fig. 2a) and electron backscatter diffraction (EBSD) results (Fig. 2b–d) show that the as-annealed Ni10 and Ni5 alloys were single-phase FCC solid solutions with an average grain size of ~100 μm, while the as-annealed Ni0 alloy exhibited a dual-phase FCC-HCP structure with an HCP phase fraction of ~40 vol.% from EBSD analysis.

### Reversible martensitic transformation

**Figure 3:**
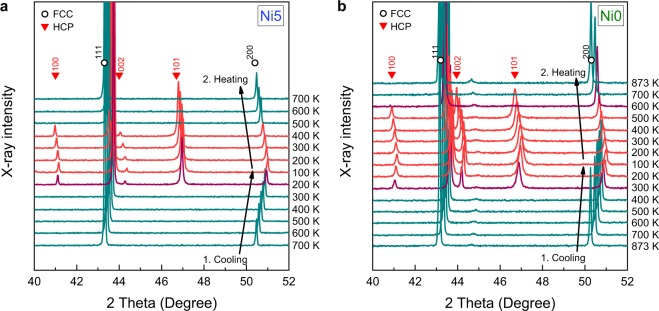
*In situ* XRD patterns at various temperatures. (**a**) XRD patterns of as-annealed Ni5 alloy between 100 and 700 K. The initial XRD pattern at 700 K corresponds to an FCC single-phase structure. New peaks, corresponding to the HCP phase, appear from 200 K during cooling and fully revert to the initial FCC peaks at 500 K during heating. (**b**) XRD patterns of as-annealed Ni0 alloy between 100 K and 873 K. The initial pattern at 873 K corresponds to a FCC single-phase structure, but shows a small peak around 45°. This peak likely corresponds to the Mn_2_O_3_ phase, which is one of the oxides that forms on the surface of Cr_20_Mn_20_Fe_20_Co_20_Ni_20_ alloy at 873 K^33^. HCP peaks appear from 300 K during cooling and revert to the FCC peaks at 700 K during heating. Shifts of all peaks to higher angles during cooling and to lower angles during heating result from the contraction and expansion of the unit cells, respectively.

Figure 3a shows the XRD patterns of the Ni5 alloy collected in the temperature range from 100 to 700 K. The alloy was initially heated to 700 K under vacuum, then the XRD patterns were obtained during cooling to 100 K and then subsequent heating to 700 K. Compared to the Ni10 alloy, which was a single-phase FCC solid solution during cooling down to 100 K (Figure S1), the Ni5 alloy exhibited diffraction peaks of the HCP phase when the temperature decreased to 200 K. With further cooling to 100 K, the HCP peaks grew in intensity while those from the initial FCC phase decreased. The HCP peaks remained stable during heating to 400 K, though the HCP phase reverted to the initial FCC phase with heating to 500 K. The unit cell parameters were determined from the XRD pattern at 300 K to be *a* = 3.58747 ± 0.00001 Å for the FCC phase and *a* = 2.5369 ± 0.0004 Å and *c* = 4.0944 ± 0.0018 Å for the HCP phase. The Ni5 alloy cooled by liquid nitrogen exhibited a dual-phase FCC–HCP structure with a thermally induced HCP phase fraction of ~20 vol.% from EBSD analysis and a homogeneous elemental distribution confirmed by electron probe microanalysis (Figure S2). These results reveal a reversible martensitic transformation between the FCC and HCP phases in the Ni5 alloy.

A similar phase transition was observed in the Ni0 alloy (Fig. 3b). The alloy showed higher onset temperatures, where the HCP phase appears (300 K) and disappears (700 K), than the Ni5 alloy, indicating an enhanced HCP phase stability, as predicted in Fig. 1c. The unit cell parameters were calculated to be *a* = 3.58528 ± 0.00001 Å for the FCC phase and *a* = 2.5363 ± 0.001 Å and *c* = 4.093 ± 0.005 Å for the HCP phase at 300 K. A small peak around 45°, observed after the initial heating to 873 K, is likely to correspond to a Mn_2_O_3_ phase, which is one of the oxides formed on the surface of Cr_20_Mn_20_Fe_20_Co_20_Ni_20_ HEA at 873 K^33^.

**Figure 4:**
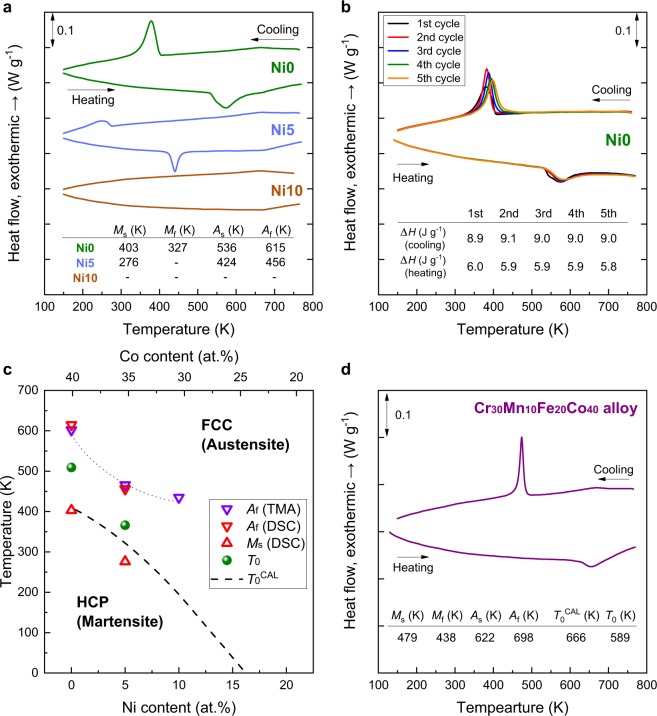
Martensitic transformation in 3*d* HEAs. (**a**) DSC curves of as-annealed Ni0, Ni5, and Ni10 alloys. The transformation temperatures determined from the curves are summarized. (**b**) DSC curves of as-annealed Ni0 alloys obtained for five cooling and heating cycles. (**c**) Variation of the manrtensitic transformation temperatures (*M*_s_ and *A*_f_) and thermodynamic equilibrium temperature (*T*_0_ = (*M*_s_ + *A*_f_)/2 and *T*_0_^CAL^ (dashed line)) as a function of Ni content in the Cr_20_Mn_20_Fe_20_Co_40-x_Ni_x_ system. *A*_f_ determined by a thermo-mechanical analyzer (Figure S3) is included for comparison. The increase in *T*_0_ with a decrease in the Ni/Co ratio is well matched with the increase in *T*_0_^CAL^. (**d**) A DSC curve of as-annealed Cr_30_Mn_10_Fe_20_Co_40_ alloy.

Figure 4a shows the results of thermal analysis of the Ni10, Ni5, and Ni0 alloys measured by differential scanning calorimetry (DSC). An exothermic peak during cooling and an endothermic peak during heating were observed for the Ni5 and Ni0 alloys, indicating forward and reverse martensitic transformations, respectively. The martensitic transformation start temperature (*M*_s_), martensitic transformation finish temperature (*M*_f_), reverse transformation start temperature (*A*_s_), and reverse transformation finish temperature (*A*_f_) obtained from the DSC curves are summarized in Fig. 4a. The onset temperatures of the martensitic transformation measured by *in situ* XRD analysis match well with those measured by DSC analysis. Figure 4b shows the DSC results of Ni0 alloy in five consecutive cycles. A slight increase in *M*_s_ by the cycling was observed but the transformation heats for forward (~9.0 J·g^−1^) and reverse transformation (~6.0 J·g^−1^) were very stable as compared to Co-32Ni alloy where the transformation peak rapidly decayed by thermal cycling^34^. Thus, the martensitic transformation is highly reversible in the present alloys.

We compared the characteristic temperatures of martensitic transformation measured by DSC and a thermo-mechanical analyzer (Figure S3) with *T*_0_^CAL^ as a function of Ni content in the Cr_20_Mn_20_Fe_20_Co_40-x_Ni_x_ system (Fig. 4c). The thermodynamic equilibrium temperature (*T*_0_ = (*M*_s_ + *A*_f_)/2) was calculated from the DSC results to be 366 and 509 K for the Ni5 and Ni0 alloys, respectively. The predicted *T*_0_^CAL^ monotonically increased with a decrease in the Ni/Co ratio, and an increasing trend was also seen in the experimentally determined *T*_0_. In addition to the Ni5 and Ni0 alloys, the Cr_30_Mn_10_Fe_20_Co_40_ alloy, which was predicted to show the lowest Δ*G*^HCP-FCC^ (Fig. 1b) and the highest *T*_0_^CAL^ (Fig. 1d), exhibited higher *M*_s_ (479 K), *A*_f_ (698 K), and *T*_0_ (589 K) than the Ni0 alloy (Fig. 4d). These results validate the CALPHAD prediction in Fig. 1 that the HCP phase stability as well as *T*_0_ can be tailored through composition tuning in 3*d* HEAs.

### Shape memory effect

**Figure 5:**
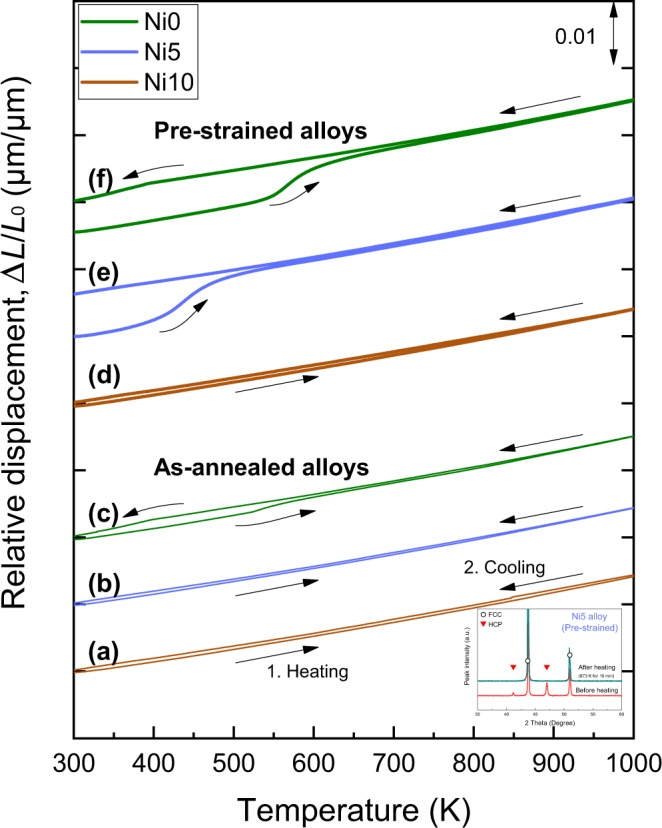
Shape memory effect of 3*d* HEAs. (**a**–**c**) Relative change in specimen length (Δ*L*/*L*_0_, Δ*L* is the change in length and *L*_0_ is the initial length) during heating to 1000 K and cooling to room temperature for as-annealed (**a**) Ni10, (**b**) Ni5, and (**c**) Ni0 alloys. Compared with the Ni10 and Ni5 alloys, the Ni0 alloy exhibits a dilation and contraction during heating and cooling, respectively. (**d**–**f**) Δ*L*/*L*_0_ for pre-strained (**d**) Ni10, (**e**) Ni5, and (**f**) Ni0 alloys with a compressive strain of ~1% deformed at room temperature. Compared with the Ni10 alloy, the Ni5 and Ni0 alloys exhibit a drastic dilation during heating. The marked dilation leads to a pronounced increase in specimen length after cooling, demonstrating the shape memory effect of Ni5 and Ni0 alloys. XRD patterns of the pre-strained Ni5 alloy (inset) show an FCC/HCP dual-phase structure before heating but an FCC single-phase structure after heating to 873 K for 10 min, indicating that the shape memory effect is associated with the martensitic transformation between the FCC and HCP phases.

Figure 5a–c shows the relative change in the specimen length (Δ*L*/*L*_0_, where Δ*L* is the change in length and *L*_0_ is the initial length) of the as-annealed Ni10, Ni5, and Ni0 alloys measured by a thermo-mechanical analyzer. The as-annealed Ni10 and Ni5 alloys exhibited thermal expansion curves similar to the single-phase Cr_20_Mn_20_Fe_20_Co_20_Ni_20_ HEA^35^, indicating that no phase transformation occurred during heating to 1000 K and cooling to room temperature. However, the as-annealed Ni0 alloy displayed a dilation during heating and a contraction during cooling. The onset temperatures of the dilation and contraction were 531 and 399 K, respectively.

Figure 5d–f shows the thermal expansion and contraction curves of the pre-strained Ni10, Ni5, and Ni0 alloys deformed with a compressive strain of about 1% at room temperature. In contrast to the pre-strained Ni10 alloy, the pre-strained Ni5 and Ni0 alloys displayed a significant dilation during heating and the pre-strained Ni0 alloy showed a contraction during cooling. The onset temperatures of the dilation for Ni5 and Ni0 alloys and the contraction for Ni0 alloy were 402, 542, and 397 K, respectively. The three as-annealed alloys and the pre-strained Ni10 alloy exhibited a small increase in Δ*L*/*L*_0_ of less than 5 × 10^−4^ (corresponding to 5 μm in specimen length) after cooling. However, the increase in Δ*L*/*L*_0_ of the pre-strained Ni5 and Ni0 alloys was more than ten times larger than that of the other alloys. The significant increase in specimen length can be attributed to the marked dilation during heating, revealing the shape memory effect of Ni5 and Ni0 alloys.

The Ni10 alloy with a single-phase structure showed no phase transformation, whereas the Ni0 alloy with a dual-phase structure displayed a distinct dilation during heating and contraction during cooling in both the as-annealed and pre-strained states. No phase transformation was observed for the as-annealed Ni5 alloy, but marked dilation was observed for the pre-strained Ni5 alloy. XRD results revealed that the pre-strained Ni5 alloy showed a dual-phase structure while the alloy after heating to 873 K for 10 min showed a single-phase structure (inset in Fig. 5). The onset temperatures of the dilation and contraction in the dual-phase alloys (Fig. 5c,e,f) agreed well with *A*_s_ and *M*_s_ measured by DSC analysis, indicating that the dilation and contraction are associated with the reverse and forward martensitic transformations between the FCC and HCP phases, respectively.

The dilation observed during heating was stronger in the pre-strained Ni5 and Ni0 alloys than the as-annealed Ni0 alloy. The small dilation in the as-annealed Ni0 alloy was due to the reverse martensitic transformation of the thermally induced HCP phase^31^. The significant dilation in the pre-strained Ni5 and Ni0 alloys originated from the reverse martensitic transformation of the stress-induced HCP phase. When the initial FCC phase of the Ni5 and Ni0 alloys was strained, the HCP phase was stress-induced by the movement of Shockley partial dislocations preferentially in the direction of the applied shear stress^36^. The reverse martensitic transformation of the stress-induced HCP phase was achieved by the reverse motion of the partial dislocations along this direction^36,37^. Thus, the shape recovery of the Ni5 and Ni0 alloys can be attributed to the formation of the stress-induced HCP phase during a shape change and the reverse martensitic transformation of the stress-induced HCP phase on heating.

### Recovery strain

**Figure 6:**
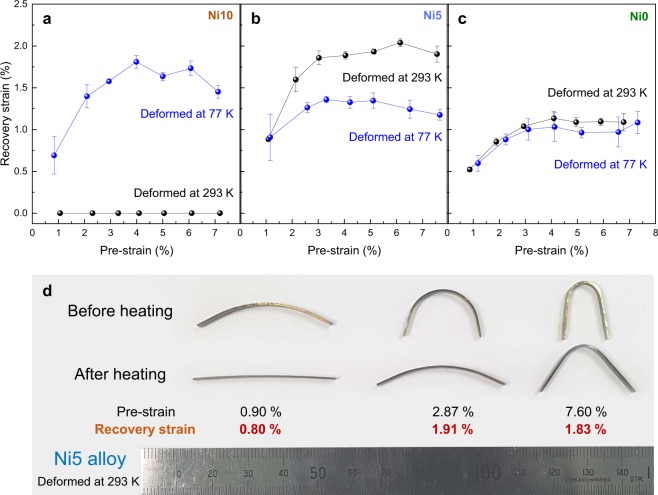
Recovery strain of 3*d* HEAs. (**a**–**c**) Recovery strain in (**a**) Ni10, (**b**) Ni5, and (**c**) Ni0 alloys measured by a bending test at 293 and 77 K, respectively. All three alloys clearly exhibit a shape memory effect when deformed at both temperatures except for the Ni10 alloy deformed at 293 K. (**d**) Appearance of the deformed Ni5 alloys at 293 K before and after heating to 873 K for 10 min. The pre-strain and recovery strain of each specimen are marked in the figure.

Figure 6a–c shows the recovery strain in the Ni10, Ni5, and Ni0 alloys measured by bending rectangular specimens with dimensions of 3 × 0.6 × 40 mm^3^ at two different deformation temperatures, room (293 K) and liquid-nitrogen (77 K) temperatures. The Ni10 alloy deformed at 293 K exhibited no recovery strain, while the alloy deformed at 77 K showed a recovery strain with a maximum value of ~1.8%. The Ni5 and Ni0 alloys at both temperatures exhibited recovery strain with maximum values of ~2.0% and ~1.1% when deformed at 293 K and ~1.4% and ~1% when deformed at 77 K, respectively. The Ni5 alloy deformed at 293 K displayed the largest recovery strain among the alloys (Fig. 6d and Supplementary Video 1). The recovery strain in the alloys increased with an increase in the pre-strain, but remained stable after reaching a pre-strain of ~3.0%.

With a decrease in deformation temperature from 293 to 77 K, the recovery strain decreased by ~30% for the Ni5 alloy. The alloy showed a dual-phase structure with an HCP fraction of ~20 vol.% after cooling to 77 K (Figure S2). Since the thermally induced HCP phase suppresses the stress-induced martensitic transformation^20^ and the stress-induced HCP phase interacts with the pre-existing phase boundaries during pre-straining^32^, the stress-induced martensitic transformation and the reverse martensitic transformation of the stress-induced HCP phase are interrupted in the dual-phase alloys. Thus, the Ni5 alloy exhibited a smaller recovery strain when deformed at 77 K. The reason why the recovery strain is almost the same in the Ni0 alloy deformed at both temperatures is unclear, but the smaller recovery strain in the Ni0 alloy than the Ni5 alloy may be associated with the higher fraction (~40 vol.%) of the thermally induced HCP phase at room temperature (Fig. 2d).

The Ni10 alloy exhibited the largest recovery strain among the alloys deformed at 77 K. The pre-strained Ni10 alloy at 77 K exhibited distinct dilation during heating in the thermo-mechanical analysis (Figure S3), indicating a reverse martensitic transformation from the HCP to FCC phase. However, a forward martensitic transformation was not observed in the *in situ* XRD (Figure S1), DSC analysis (Fig. 4a), and the XRD pattern of the alloy cooled by liquid nitrogen (not shown), implying that *M*_s_ of the Ni10 alloy was lower than 77 K. Thus, the Ni10 alloy was predominantly deformed by the stress-induced martensitic transformation at 77 K without any thermally induced HCP phase, which is one of the reasons why the Ni10 alloy deformed at 77 K exhibited a similar recovery strain as the Ni5 alloy deformed at 293 K.

## Discussion

In this work, we have developed novel shape memory alloys in the CrMnFeCoNi alloy system using the CALPHAD methodology by calculating the Δ*G*^HCP-FCC^ variation for a wide composition range of 3*d* HEAs. In comparison with the previously reported SMAs with martensitic transformation between the FCC and HCP phases, the recovery strain in the Ni5 alloy deformed at 293 K was found to be much larger than that in CoNi^34,38–40^, FeMn^41^, and FeMnC^42^ alloys (less than 0.3%^38,42^) and comparable to that in polycrystalline FeMnSi-based alloys (~2.0%^41^) containing Si up to about 11 at.%^43,44^, used for industrial applications like pipe joining and seismic damping^44,45^. For CoNi and FeMn binary alloys with a poor shape memory effect, the addition of a significant amount of Si led to a remarkable improvement in the recovery strain. The roles of Si in the shape memory effect have been determined to be^41–46^ 1) suppressing the magnetic transition from paramagnetism to anti-ferromagnetism, which inhibits the martensitic transformation due to magnetic ordering, 2) enhancing the reversibility of the martensitic transformation by decreasing the volume change between the FCC and HCP phases, and 3) solid-solution strengthening of the FCC phase to suppress the glide motion of dislocations.

The Cr_20_Mn_20_Fe_20_Co_20_Ni_20_ alloy was predicted to show a very low magnetic transition temperature of 23 K by the addition of Cr and Mn^47^, implying that the martensitic transformation is not strongly affected by the magnetic transition in the non-equiatomic CrMnFeCoNi HEAs. The volume changes between the FCC and HCP phases calculated from the lattice constants^46^ were 1.15 ± 0.07% and 1.05 ± 0.2% for the Ni5 and Ni0 alloys, respectively. These values are much lower than the values of 2.26% for the Fe_74.5_Mn_24.5_ alloy and 1.42% for the Fe_65.5_Mn_25.1_Si_9.4_ alloy^46^, which indicates an enhanced reversibility of the martensitic transformation in the Ni5 and Ni0 alloys without the addition of Si. The yield strengths of the Cr_20_Mn_20_Fe_20_Co_20_Ni_20_ alloy (from 160 MPa^16^ to 260 MPa^11^ for a grain size from 140 to 16 μm, respectively) and the Ni5 alloy (218 MPa for a grain size of 18 μm, not shown) were comparable to that of the Fe_71.5_Mn_16.1_Si_11.1_C_1.3_ alloy (200 MPa^42^) but much higher than that of the Fe_75.7_Mn_24.3_ (80 MPa^41^) and Co_68.9_Ni_31.1_ (85 MPa^38^) alloys. The higher yield strength resulted from the enhanced solid solution strengthening of the HEA, which contributes to the stress-induced martensitic transformation as a predominant deformation mode in the pre-straining. Therefore, the large recovery strain in non-equiatomic CrMnFeCoNi HEAs with shape memory effect can be attributed to the reduced volume change between the FCC and HCP phases and the improved yield strength of the FCC phase.

**Figure 7:**
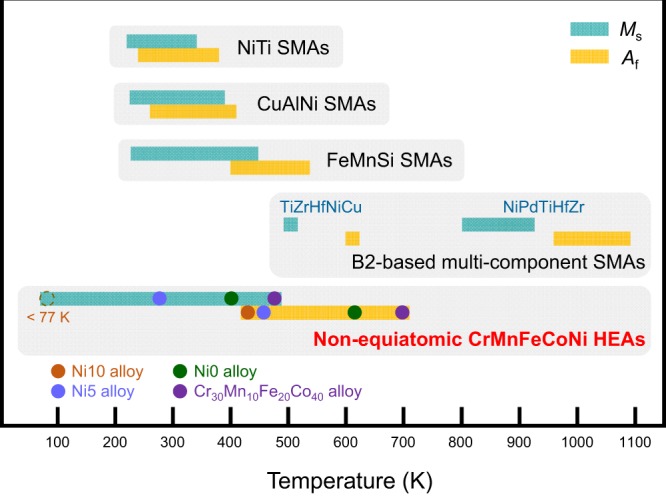
Comparison of martensitic transformation temperatures. The characteristic temperature range of martensitic transformation (*M*_s_ and *A*_f_) in non-equiatomic CrMnFeCoNi HEAs (this study) and B2-based multi-component SMAs (TiZrHfNiCu^24^ and NiPdTiHfZr^25^ alloys), compared with the temperature range in conventional SMAs such as binary TiNi^29^, ternary CuAlNi^30^, and ternary FeMnSi^31,32^. Non-equiatomic CrMnFeCoNi HEAs exhibit a wider range and higher limit of *M*_s_ and *A*_f_ than the conventional SMAs.

Figure 7 displays the characteristic temperature range of martensitic transformation (*M*_s_ and *A*_f_) in non-equiatomic CrMnFeCoNi HEAs with shape memory effect (Fig. 4) and B2-based multicomponent SMAs (TiZrHfNiCu^24^ and NiPdTiHfZr^25^ alloys), compared with the characteristic temperature range in conventional SMAs such as binary TiNi, ternary CuAlNi, and ternary FeMnSi^29–32^. Interestingly, similar to the B2-based multi-component SMAs, some of the developed non-equiatomic CrMnFeCoNi HEAs exhibit much higher *M*_s_ than 373 K, which is regarded as an upper limit of *M*_s_ in commercial SMAs like binary TiNi alloys^48,49^. Also, the developed non-equiatomic CrMnFeCoNi HEAs exhibit a wider range of transformation temperatures, e.g., from less than 77 to 479 K for *M*_s_ and from 435 to 698 K for *A*_f_ than the conventional SMAs. These findings suggest that developed non-equiatomic CrMnFeCoNi HEAs, which have excellent workability, weldability, fracture resistance, and corrosion resistance^12,50–52^, are promising candidates for shape memory actuators at elevated temperatures. It should be noted that much higher *M*_s_ and *A*_f_ could be achieved in the CrMnFeCoNi alloy system by exploring a broad range of compositions where FCC phase is predicted to be formed^28^. For the high-temperature applications, the thermal stability of FCC phase at the elevated temperatures is a major issue. Reduced transformation hysteresis (*A*_f_*–M*_s_) would be preferred for actuator applications. In general transformation hysteresis is related to the mobility of austenite/martensite interface and more glissile interface may lead to smaller hysteresis. These subjects are currently under investigation.

In conclusion, we have developed novel 3*d* HEAs with shape memory effect in the CrMnFeCoNi alloy system. The designed non-equiatomic CrMnFeCoNi HEAs displayed thermally- and stress-induced martensitic transformations between the FCC and HCP phases. The appreciable shape memory properties, comparable to those of polycrystalline FeMnSi-based alloys, result from the reverse martensitic transformation of the stress-induced HCP phase, which is predominantly assisted by the enhanced solid solution strengthening of the FCC phase and reversibility of the martensitic transformation between the FCC and HCP phases. The highest *M*_s_ and *A*_f_ in the developed non-equiatomic CrMnFeCoNi HEAs were higher than those in conventional SMAs, and these shape recovery temperatures could be made even higher via customized compositional manipulation of the developed HEAs with shape memory effect in a wide composition range. These findings allow us to use the non-equiatomic CrMnFeCoNi HEAs as a new class of potential high-temperature SMAs and offer new insights on how to develop novel HEAs with customized shape memory effect.

## Methods

### Materials processing

Ingots of a series of Cr_20_Mn_20_Fe_20_Co_40-x_Ni_x_ (x = 0, 5, 10, and 20 at.%) and Cr_30_Mn_10_Fe_20_Co_40_ alloys, 1 kg in weight, were produced by high frequency vacuum induction melting. The as-cast ingots with cross-sections of 30 × 30 mm^2^ were homogenized at 1473 K for 24 h under Ar atmosphere followed by water quenching. The homogenized ingots were subjected to multi-pass caliber rolling at 673 K to form squared bars^53^ with cross-sections of 14 × 14 mm^2^, then annealed at 1373 K for 1 h.

### Microstructural characterization

The phase constitutions in the temperature ranges 100–700 K for the Ni5 alloy and 100–873 K for the Ni0 alloy were confirmed by low- and high-temperature XRD experiments (SmartLab diffractometer with TTK-600 chamber, Rigaku, Tokyo, Japan) using a Cu *K*α_1_ X-ray source at 45 kV and 200 mA under vacuum. For the XRD analysis, the surfaces of the as-annealed specimens were pre-etched using aqua-regia (3HCl:1HNO_3_) solution to remove the oxidized surface. Differential scanning calorimetry (DSC; DSC Q2000, TA Instruments, New Castle, DE, USA) analysis was conducted with a constant cooling/heating rate of 20 K·min^−1^. The microstructure was examined by a scanning electron microscope (SEM; JSM-7001F, JEOL, Tokyo, Japan) equipped with EBSD. The EBSD analysis was performed at an accelerated voltage of 20 kV and step size of 1 μm. Electron probe microanalysis (JXA-8500F, JEOL Ltd, Tokyo, Japan) was conducted to analyze the elemental distribution of the alloys.

### Evaluation of the shape memory effect

A thermal expansion test of the as-annealed and pre-strained specimens was performed using a thermo-mechanical analyzer (TMA; Q400, TA Instruments, New Castle, DE, USA) at a constant heating/cooling rate of 5 K·min^−1^ with a specimen dimension of 4 × 4 × 10 mm^3^. The pre-strained specimens with a strain of about 1% were prepared by compressive tests at a strain rate of 1 × 10^−3^ s^−1^. The recovery strain was evaluated by a bending test at room and liquid-nitrogen temperatures. Specimens with dimensions of 3 × 0.6 × 40 mm^3^ were bent using steel pieces with different radii. For the bending test at liquid-nitrogen temperature, the specimens were immersed in liquid nitrogen for 10 min and bent using stainless steel tweezers. The bending strains in the deformed specimens before and after heating to 873 K for 10 min were calculated from the radius of curvature ( = *t*/2*r* × 100, where *t* is the specimen thickness and r is the radius of curvature) measured by an optical microscope with image analysis software (Figure S4).

## Supplementary information

Supplementary information video

Supplementary information figures

## Data Availability

The data that support the findings of this study are available from the corresponding authors, at jilee@pusan.ac.kr or Tsuchiya.Koichi@Nims.go.jp, upon reasonable request.
