# Supplementary material for: A strategy of designing high-entropy alloys with high-temperature shape memory effect
Source: Sci Rep. 2019 Sep 11;9:13140. doi: 10.1038/s41598-019-49529-8 (PMC6739314; doi:10.1038/s41598-019-49529-8)
Supplement: Supplementary file 2 — Supplementary information figures [file 41598_2019_49529_MOESM2_ESM.pdf]

Supplementary Information

A strategy of designing high-entropy alloys with high-temperature shape memory effect

J. I. Lee, K. Tsuchiya, W. Tasaki, H. S. Oh, T. Sawaguchi,
H. Murakami, Takanobu Hiroto, Y. Matsushita, and E. S. Park

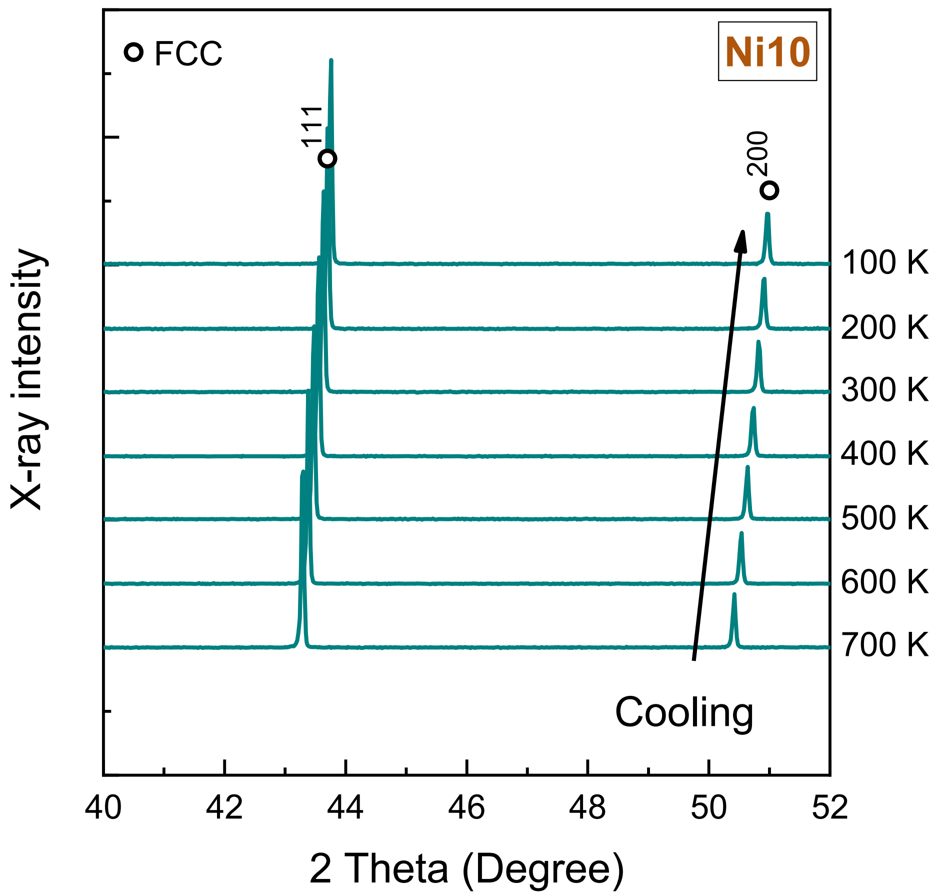

Supplementary Figure 1 | *In-situ* XRD patterns of as-annealed Ni10 alloy at various temperatures. The Ni10 alloy exhibits a FCC single-phase structure during cooling down to 100 K. All the peaks shift to higher angles during cooling due to the contraction of the unit cells.

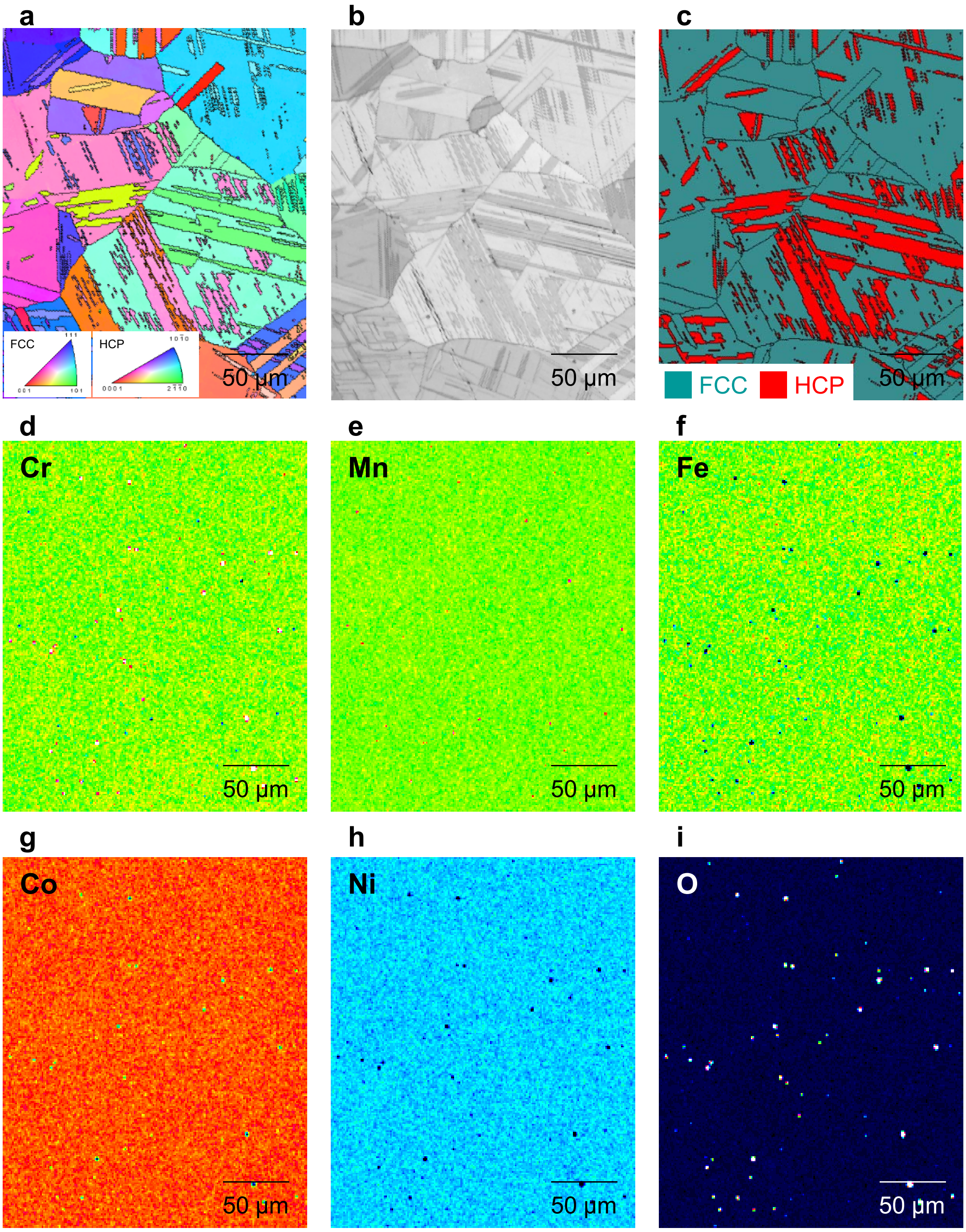

Supplementary Figure 2 | Microstructure of the as-annealed Ni5 alloy cooled by liquid nitrogen. (**a**) EBSD IPF, (**b**) IQ, and (**c**) phase map, showing FCC/HCP dual-phase structure with HCP fraction of about 20 %. EPMA elemental maps of (**d**) Cr (**e**) Mn, (**f**) Fe, (**g**) Co, (**h**) Ni, and (**i**) O, displaying the homogeneous distribution of the five principal elements in the dual-phase structure and the dispersion of oxide particles, which correspond to $MnCr_2O_4$.

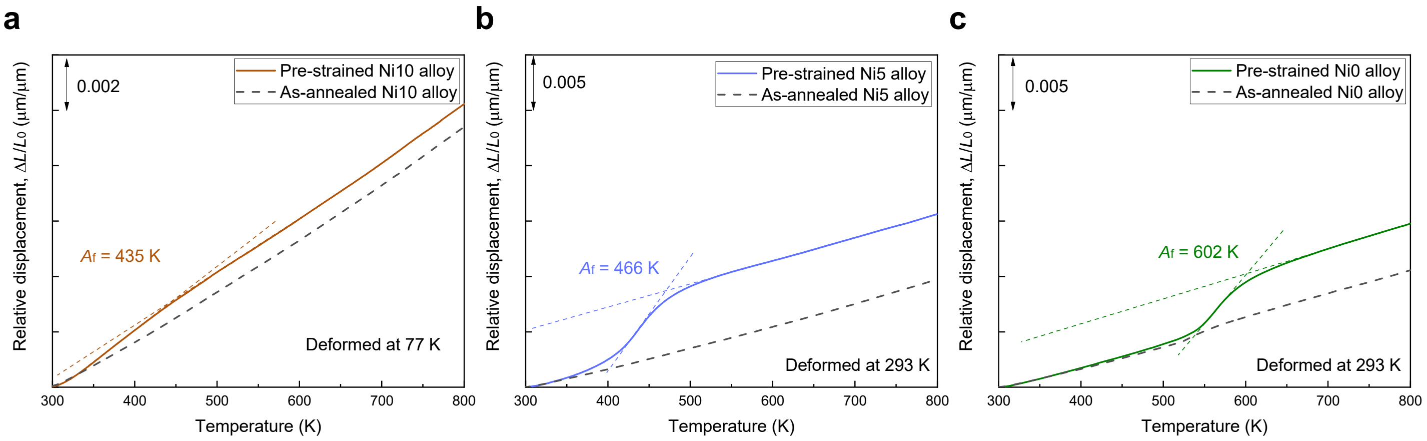

Supplementary Figure 3 | Relative change in specimen length during heating. (**a**) Thermal expansion curves of pre-strained Ni10 alloy with a compressive strain of ~ 1 % deformed at 77 K. (**b-c**) Thermal expansion curves of the pre-strained (**b**) Ni5 and (**c**) Ni0 alloys with a compressive strain of ~ 1 % deformed at room temperature. Thermal expansion curves of the as-annealed alloys are shown as dashed lines for comparison.

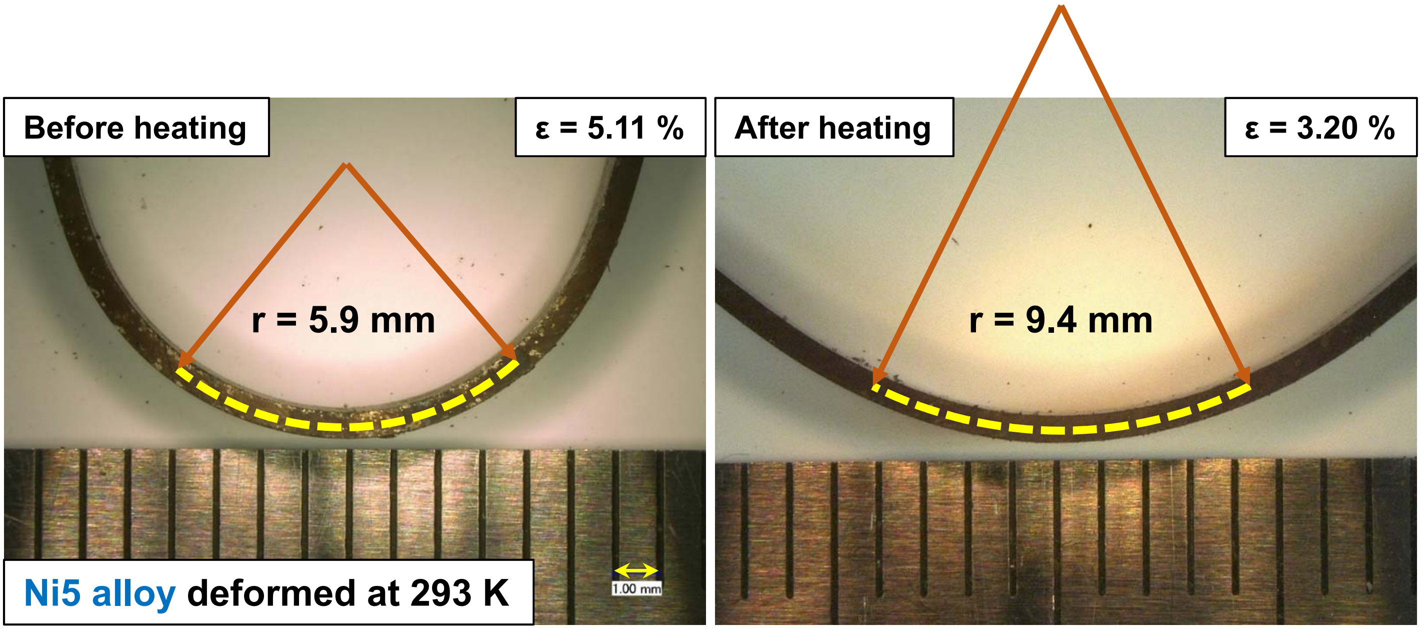

Supplementary Figure 4 | Evaluation of recovery strain in Ni5 alloy deformed at 293 K. Recovery strain calculated from the difference in the bending strains before and after heating to 873 K for 10 min. The radius of curvature in the central area in the central area of the pre-strained specimens was measured by optical microscope equipped with image analysis software.
